# Personalized Drug Screening and Risk Assessment in Patient-Derived Gastroenteropancreatic Neuroendocrine Neoplasms

**DOI:** 10.1210/clinem/dgaf705

**Published:** 2026-01-10

**Authors:** Christoph J Auernhammer, Katharina Wang, Umberto Maccio, Thomas Knösel, Maximilian P Hungbauer, Katharina Schilbach, Julian Maurer, Lea Peischer, Astrid Reul, Elena Kuzmenko, Edlira Luca, Julia Hamati, Diana Vetter, Jose Oberholzer, Ralph Fritsch, Karel Pacak, Ashley B Grossman, Felix Beuschlein, Martin Reincke, Constanze Hantel, Kathrin Zitzmann, Svenja Nölting

**Affiliations:** Department of Medicine IV, LMU University Hospital, LMU Munich, 80336 Munich, Germany; Interdisciplinary Center of Neuroendocrine Tumors of the GastroEnteroPancreatic System (GEPNET-KUM, ENETS Centre of Excellence), LMU University Hospital, 81377 Munich, Germany; Department of Medicine IV, LMU University Hospital, LMU Munich, 80336 Munich, Germany; Department of Pathology and Molecular Pathology, University Hospital Zurich, Zurich CH-8091, Switzerland; Interdisciplinary Center of Neuroendocrine Tumors of the GastroEnteroPancreatic System (GEPNET-KUM, ENETS Centre of Excellence), LMU University Hospital, 81377 Munich, Germany; Institute of Pathology, Faculty of Medicine, LMU Munich, 80337 Munich, Germany; Interdisciplinary Center of Neuroendocrine Tumors of the GastroEnteroPancreatic System (GEPNET-KUM, ENETS Centre of Excellence), LMU University Hospital, 81377 Munich, Germany; Department of General, Visceral and Transplantation Surgery, LMU University Hospital, 81377 Munich, Germany; Department of Medicine IV, LMU University Hospital, LMU Munich, 80336 Munich, Germany; Department of Medicine IV, LMU University Hospital, LMU Munich, 80336 Munich, Germany; Department of Medicine IV, LMU University Hospital, LMU Munich, 80336 Munich, Germany; Department of Endocrinology, Diabetology and Clinical Nutrition, University Hospital Zurich, Zurich CH-8091, Switzerland; Department of Endocrinology, Diabetology and Clinical Nutrition, University Hospital Zurich, Zurich CH-8091, Switzerland; Department of Endocrinology, Diabetology and Clinical Nutrition, University Hospital Zurich, Zurich CH-8091, Switzerland; Department of Medicine IV, LMU University Hospital, LMU Munich, 80336 Munich, Germany; Department of Visceral and Transplantation Surgery, University Hospital Zurich, Zurich CH-8091, Switzerland; Department of Visceral and Transplantation Surgery, University Hospital Zurich, Zurich CH-8091, Switzerland; Department of Medical Oncology and Hematology, University Hospital Zurich, Zurich CH-8091, Switzerland; Center for Adrenal Endocrine Tumors, AKESO, Prague 5, Czech Republic; Faculty of Medicine, Palacky University, 779 00 Olomouc, Czech Republic; Green Templeton College, University of Oxford, Oxford OX2 6HG, UK; NET Unit, ENETS Centre of Excellence, Royal Free Hospital, London NW3 2QG, UK; Department of Medicine IV, LMU University Hospital, LMU Munich, 80336 Munich, Germany; Department of Endocrinology, Diabetology and Clinical Nutrition, University Hospital Zurich, Zurich CH-8091, Switzerland; The LOOP Zurich–Medical Research Center, Zurich CH-8044, Switzerland; ENETS Centre of Excellence Zurich, University Hospital Zurich, Zurich CH-8091, Switzerland; Department of Medicine IV, LMU University Hospital, LMU Munich, 80336 Munich, Germany; Interdisciplinary Center of Neuroendocrine Tumors of the GastroEnteroPancreatic System (GEPNET-KUM, ENETS Centre of Excellence), LMU University Hospital, 81377 Munich, Germany; Department of Endocrinology, Diabetology and Clinical Nutrition, University Hospital Zurich, Zurich CH-8091, Switzerland; Department of Medicine IV, LMU University Hospital, LMU Munich, 80336 Munich, Germany; Department of Medicine IV, LMU University Hospital, LMU Munich, 80336 Munich, Germany; Department of Endocrinology, Diabetology and Clinical Nutrition, University Hospital Zurich, Zurich CH-8091, Switzerland; ENETS Centre of Excellence Zurich, University Hospital Zurich, Zurich CH-8091, Switzerland

**Keywords:** neuroendocrine tumor, primary cultures, precision medicine, personalized drug testing

## Abstract

**Context:**

Precision medicine has transformed many areas in oncology. However, it remains largely unexplored in metastatic gastroenteropancreatic neuroendocrine neoplasms (GEP-NENs), where there is a need for further innovative therapies.

**Objective:**

To evaluate individual tumor responses to different agents, we have established a standardized personalized drug screening and risk assessment platform using patient-derived GEP-NEN primary cultures (n = 23, 16/23 from metastatic tumors, n = 12 small intestinal neuroendocrine tumors [siNETs], n = 10 pancreatic NETs [pNETs], n = 1 neuroendocrine carcinoma [NEC]).

**Methods:**

We assessed GEP-NEN primary culture cell viability, performed signaling pathway analysis by automated Western blotting and immunohistochemically evaluated tumor composition.

**Results:**

Systematic drug testing of 27 agents including signaling inhibitors (i) (mechanistic target of rapamycin inhibitor [mTORi] everolimus, tyrosine kinase inhibitors cabozantinib/sunitinib, AKTi capivasertib, PI3Ki alpelisib, CDK4/6i ribociclib), DNA damage response inhibitors (PARPi niraparib, WEE1i adavosertib, ATRi berzosertib), chemotherapeutics (temozolomide, 5-fluorouracil, lurbinectedin), drug repurposed agents (zoledronic acid), and a personalized risk assessment (glucagon-like peptide [GLP]-2 analogue teduglutide, GLP-1 analogue semaglutide, sex hormones) was performed. We demonstrated statistically significant group effects and individualized responsiveness/resistance data. We identified differences in drug response between pNETs/siNETs and between GEP-NETs/GEP-NEC, respectively.

**Conclusion:**

We provide novel data on the efficacy of putative and established therapies in patient-derived GEP-NEN primary cultures. Our standardized platform for personalized drug screening and risk assessment in GEP-NEN primary cultures enables prediction of individual tumor treatment response in this orphan disease.

Precision medicine tailored to individual patients is already established in many oncological disciplines, but is yet only emerging in the field of gastroenteropancreatic (GEP) neuroendocrine neoplasms (NENs) (1). GEP-NENs can be stratified into well-differentiated neuroendocrine tumors (NETs) and poorly differentiated neuroendocrine carcinomas (NECs) (2). NETs are further classified as G1, G2, or G3 based on proliferative markers, while NECs can be differentiated by small or large cell subtypes (3).

The survival of GEP-NET and GEP-NEC patients largely depends on tumor localization, grading, and TNM stage. While 5-year survival of localized GEP-NET (G1-G2) and GEP-NEC patients lies between 70% and 80% and 25% and 40%, respectively (4), the outcome of patients with metastatic tumors is markedly worse with a 5-year survival of 45% to 63% for metastatic GEP-NETs and 3.2% to 16.6% for metastatic GEP-NECs (5, 6). This was also confirmed in a recent study evaluating survival trends of patients with NENs in the United States (overall 5-year survival: localized 90.3% vs distant 57%) (7). Site specific 5-year survival in distant disease varied according to primary tumor location (small intestinal [si]NETs 72.2%, pancreatic [p]NETs 49.7%, gastric NETs 37.6%, and colorectal NETs 31.4%-34.7%) (7).

The poor outcome of patients with metastatic GEP-NENs is partly due to an ongoing lack of curative treatment options apart from surgery.

In GEP-NETs, somatostatin receptor (SSTR) expression status is the only personalized preselection for therapy with somatostatin analogues (octreotide, lanreotide) (8, 9) or with peptide receptor radionuclide therapy (1). Other systemic treatment options for advanced or metastatic GEP-NETs include targeted therapies (the mechanistic target of rapamycin inhibitor [mTORi] everolimus, tyrosine kinase inhibitors [TKIs] cabozantinib and sunitinib) (10-13), chemotherapy regimens (streptozotocin/5-fluorouracil, capecitabine/temozolomide) (14), which are currently applied on a nonpersonalized basis. Therefore, research on personalized and novel therapy options to advance the field of precision medicine in GEP-NENs is urgently needed. A schematic representation of the main signaling pathways in GEP-NENs and their associated (potential) therapeutic targets is shown in Fig. 1.

**Figure 1.**
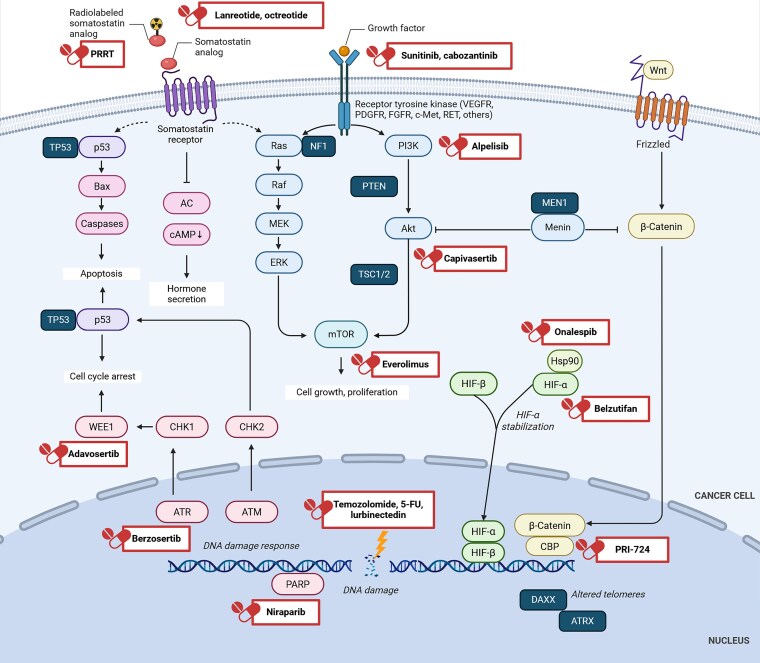
Schematic illustration of the signaling pathways in gastroenteropancreatic neuroendocrine neoplasms, their frequent alterations (dark blue) and potential therapeutic targets which were evaluated in this study. Both established and novel therapies are depicted in the red boxes.

We have established a standardized personalized drug screening and risk assessment platform for patient-derived GEP-NEN primary cultures, potentially improving informed personalized therapy decisions in these rare tumors (Fig. 2). Additionally, we exemplarily analyzed cell composition of some GEP-NEN primary cultures to ascertain the tumor cell content.

**Figure 2.**
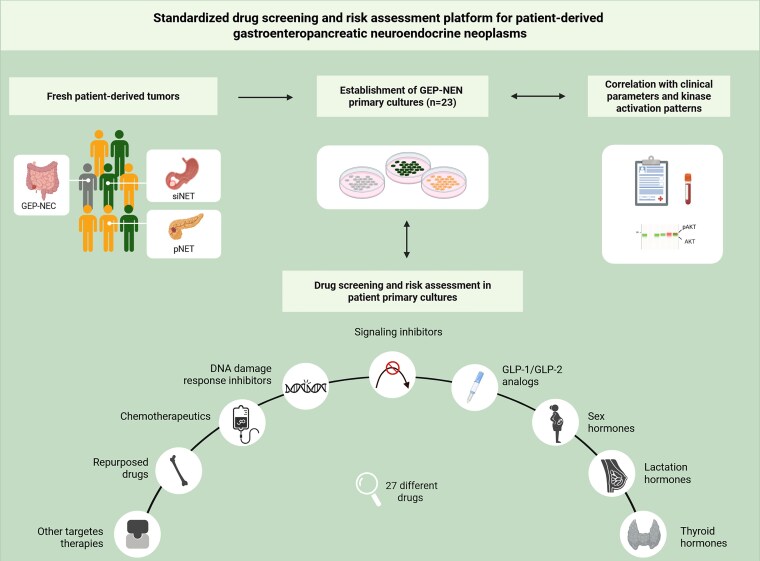
Schematic illustration of our standardized drug screening and risk assessment platform for patient-derived gastroenteropancreatic neuroendocrine neoplasms (GEP-NENs).

## Materials and methods

### Standardized protocol for establishment of gastroenteropancreatic neuroendocrine neoplasm primary cultures

This preclinical study was performed at the University Hospitals Munich and Zurich. Metastatic and nonmetastatic GEP-NEN patients undergoing surgical resection of the primary tumor, recurrence, or metastasis were included. All GEP-NEN patients who were operated on at either of the 2 centers and provided written informed consent were included in the study. Exclusion criteria were patients without consent or age younger than 18 years.

As previously described, fresh patient-derived GEP-NET tumor tissue (n = 22) was acquired from patients undergoing tumor/metastasis resection at the University Hospital Munich and Zurich to establish GEP-NET primary cultures (15, 16). Additionally, a GEP-NEC primary culture (n = 1) and a normal pancreatic tissue primary culture (n = 1) were established by the same method (17). Briefly, fresh tumor tissue/normal tissue was processed following our standard procedure by removing any adherent fatty tissue, slicing the tissue into small pieces (0.5-1 mm), centrifugation at 1400 rpm (300*g*) for 5 minutes using the UNIVERSAL 320 R general purpose centrifuge (Hettich), and incubation with collagenase type 2 (Sigma Aldrich) for around 1 hour, followed by inactivation using 2-mL fetal calf serum (Thermo Fisher Scientific). After renewed centrifugation (1400 rpm for 5 minutes), the pellet was resuspended in sterile-filtered red blood cell lysis buffer (Roche) for 7 minutes followed by another round of centrifugation (1400 rpm for 5 minutes) and resuspension in RPMI 1640 culture medium (Thermo Fisher Scientific), supplemented with 10% fetal calf serum, 1% penicillin/streptomycin (all from Thermo Fisher Scientific) and 0.4% amphotericin B (PAN-Biotech). The filtered and isolated cells were then counted and seeded into 96-well plates for adherent cells. Standard incubation times of approximately 72 hours were used to allow for cell adhesion and recovery before treatment with different drugs.

### Cell viability assay and dose finding

After primary culture establishment, the cells were incubated for 72 hours with different substances using clinically relevant concentrations or doses close to the clinically relevant concentrations. Only teduglutide was incubated for a longer duration of 7 days to reflect the long-term treatment of patients with short bowel syndrome. Dimethyl sulfoxide (DMSO; AppliChem) was used as control to account for the solvent's effects and to provide a baseline for comparison. The data are given as the percentage of control DMSO (100%). Clinically relevant concentrations were determined by identification of the mean-to-maximum plasma concentrations found in patients after treatment with the respective substance. The concentrations used in our experiments and the respective clinically-relevant concentrations are listed in Supplementary Table S1 (18). CellTiter-Blue Cell Viability Assay (Promega) of patient primary cultures was then performed using a GLOMAX plate reader (Promega), according to the manufacturer's instructions. Each experiment included 3 or 4 test values per drug concentration and patient.

### JESS simple Western

Fully automated Western blotting (JESS Simple Western; ProteinSimple) was used to analyze protein expression levels in patient-derived GEP-NET primary cultures. Whole-cell protein lysates were prepared and examined according to the manufacturer's instructions. The antibodies (Abs) used were: protein kinase B (Akt) (No. C67E7, 1:50, Cell Signaling Technology catalog No. 4691, RRID:AB_915783) and phosphorylated (p)Akt (No. D9E, 1:20, Cell Signaling Technology catalog No. 4060, RRID:AB_2315049). The secondary Ab (antirabbit horseradish peroxidase) and enhanced chemiluminescence reagents were used according to the kit's instructions (antirabbit detection module chemiluminescence; ProteinSimple, Bio-Techne). The Ab diluent, washing buffer, plates, and capillary cartridges used were derived from the 12- to 230-kDa separation module (ProteinSimple, Bio-Techne). Signal normalization was achieved using RePlex and total protein modules (RePlex reagent kit and Protein Detection Module for Chemiluminescence based total protein assays; both ProteinSimple, Bio-Techne).

### Image analysis and quantification

JESS Simple Western data were analyzed using Compass for Simple Western software (6.3.0). Images from the high dynamic range 4.0 were used for the analysis, and peaks were automatically detected. Peak areas were analyzed and phosphoprotein areas were normalized to the respective total protein areas.

### Immunohistochemical cell quantification of patient-derived tumor primary cultures

We performed immunohistochemical (IHC) cell quantification of our patient-derived primary cultures to identify the tumor composition. The primary cultures were established following our standard procedure (as described earlier). To produce cell blocks, the primary cells were centrifuged and the supernatant was removed. The cell pellet was mixed with human plasma (Biowest) and thrombin reagent (Siemens Healthineers) to induce coagulation. The clot was transferred into an embedding cassette and fixed in 4% paraformaldehyde (Morphisto). Each cell block preparation was stained with hematoxylin and eosin for a preliminary assessment of overall cellularity and sample adequacy. On average, patient-derived primary culture samples that contained more than 5000 viable cells were considered suitable for cytological evaluation. Cell quantification was performed by a board-certified pathologist (U.M.) with expertise in endocrine and neuroendocrine pathology. The assessment and cell stratification were based on the evaluation of IHC staining for 3 specific markers corresponding to the cell types of interest: synaptophysin for tumor cells, smooth muscle actin for fibroblasts, and CD45 for immune cells. Quantification was carried out by visual estimation (“eyeballing”) under a light microscope, involving the manual count of 400 cells and the determination of the percentage of cells positive for each of the 3 markers.

### Statistical analysis

Statistical analysis was performed using IBM SPSS Statistics, version 29.0.2.0 (IBM Corp, released 2023). For comparisons of treatment groups with the control group in GEP-NET patient primary cultures, including comparisons between tumor types (pancreatic NETs [pNETs] vs small intestinal NETs [siNETs]), 2-way analysis of variance followed by post hoc Bonferroni test was performed. For comparisons of treatment groups with the control group in the GEP-NEC or normal pancreatic tissue primary cultures, 2-tailed *t* test or one-way analysis of variance followed by post hoc Dunnett test was performed. A *P* value less than .05 was considered statistically significant.

Antitumor efficacy was defined as strong (>50% cell viability reduction), moderate (20%-50% cell viability reduction), and low (<20% cell viability reduction). Tumor-promoting effects were defined as any cell viability increase compared to control.

### Ethics approval and consent to participate

The use of primary tumor tissue was approved as part of NeoExNET (Exzellenz-Netzwerk für Neuroendokrine Tumoren und Hypophysen- und Nebennierenerkrankungen) by the ethics committee of LMU Munich (project No. 152-10) and by the Cantonal Ethics Committee Zurich (reference No. BASEC 2017-00950) and carried out in cooperation with the Human Tissue & Cell Research biobank. This study was conducted as secondary biobank research. Written informed consent was provided by all patients. The study was performed in accordance with the Declaration of Helsinki.

## Results

### Patient cohort and tumor composition analysis of patient-derived gastroenteropancreatic neuroendocrine neoplasm primary cultures

We established a total of 23 GEP-NEN primary cultures from 22 patients (n = 1 patient with 2 GEP-NET primary cultures). Additionally, we established 1 primary culture using normal pancreatic tissue (corresponding normal tissue to an insulinoma primary culture).

The patient characteristics are listed in Table 1. Overall, 22 GEP-NET primary cultures were included (n = 12 siNETs, n = 10 pNETs). Of these, 15 primary cultures were established from patients with metastatic disease. Additionally, one metastatic GEP-NEC primary culture was established.

**Table 1.** Patient and tumor characteristics of the primary cultures (n = 24)*^a^*

	Patient ID	Sex	Age, y	Metastatic	Site of metastases	Tumor characteristics	Ki-67	Grading	Functional
**pNET (n = 10)**	2	M	80	No	—	Primary tumor, 1 cm, SSTR2 IHC positive	<1%	NET G1	Insulinoma
	3	F	38	No	—	Primary tumor, max 3.4 cm	3%-4%	NET G2	No
	5	F	64	Yes	Lymph nodes	Metastasis (lymph node), max. 2.9 cm, capsular infiltration, ISLET1 positivity	15%	NET G2	No
	6	F	55	Yes	Liver, lymph node	Primary tumor, max. 9.2 cm, angioinvasion, infiltration of spleen and stomach, lymphatic infiltration	2%	NET G1	No
	7	M	72	Yes	Liver, lymph node	Metastasis (liver), max. 2.3 cm	5%-10%	NET G2	No
	11	M	76	Yes	Liver	Primary tumor, max. 8 mm; simultaneous bilateral renal cell carcinoma, no *VHL* pathogenic variant	3%	NET G2	No
	14	F	36	No	—	Primary tumor, 3.4 cm	1%	NET G1	No
	17	M	50	Yes	Liver, lung	Primary tumor, 3.8 cm	15%	NET G2	No
	20	M	61	Yes	Lymph node	Primary tumor, 6.5 cm, SSTR2 positive	5%	NET G1	No
	22.1	F	48	No	-	Primary tumor, 8 mm, SSTR2 positive	N/A	NET G1	Insulinoma
**Normal pancreas tissue**	22.2				-	Corresponding normal pancreatic tissue to 22.1	—	-	-
**siNET (n = 12)**	1.1	F	56	Yes	Liver, ovaries, peritoneum, lymph node	Primary tumor (ileum), max. 1.6 cm, ulceration of mucosa, muscularis propria	<2%	NET G1	Carcinoid syndrome
	1.2					Metastasis (peritoneum), max. 1.7 cm			
	4	M	58	Yes	Liver, lymph node	Metastasis (liver), max. 7.5 cm, SSTR2 IHC negative	4%	NET G2	No
	8	F	63	Yes	Liver, lymph node	Metastasis (liver), max. 7.5 cm, lymphatic infiltration	<1%	NET G1	Carcinoid syndrome
	9	M	64	Yes	Lymph node, bone	Primary tumor, max. 1.4 cm, lymphatic and perineural sheath infiltration	2%	NET G1	No
	10	M	81	Yes	Liver, lymph node	Primary tumor (ileum/cecum) max. 1.5 cm	4%	NET G2	Carcinoid syndrome
	12	M	60	No	—	Primary tumor, 0.4 cm	N/A	NET G1	No
	13	M	63	Yes	Liver, lymph nodes	Metastasis (liver), max. 2.6 cm, lymphatic infiltration; simultaneous unilateral pheochromocytoma	1%	NET G1	No
	15	M	77	No	—	Primary tumor, 2.2 cm, lymphatic infiltration	<1%	NET G1	No
	16	M	70	Yes	Lymph node	Primary tumor, max 1.6 cm	5%	NET G2	No
	18	F	55	No	—	Primary tumor, 1.1 cm, lymphatic and perineural sheath infiltration	3%	NET G2	No
	19	M	65	Yes	Lymph node	Metastasis (lymph node), 10 mm, SSTR2 positive	2%	NET G1	No
**NEC**	21	F	74	Yes	Lymph node	Primary tumor, ileocecal small cell NEC, 3.5 cm, lymphatic infiltration	>90%	NEC G3	No

Abbreviations: F, female; ID, identification; IHC, immunohistochemistry; M, male; max., maximum; N/A, not available; NEC, neuroendocrine carcinoma; NET, neuroendocrine tumor; pNET, pancreatic NET; siNET, small intestinal NET; SSTR2, somatostatin receptor 2.

^*a*^Characteristics partly published previously (15, 16).

**Figure 3.**
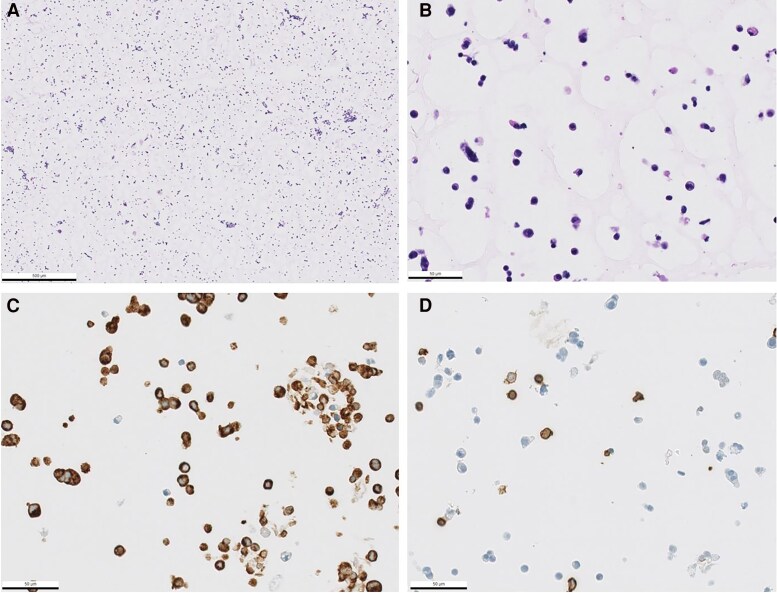
Representative image of a pancreatic neuroendocrine tumor (pNET) primary culture cell block (patient 20). A, Low-power hematoxylin and eosin staining demonstrates adequate sample cellularity. B, At higher magnification, cellular cytomorphology becomes appreciable. Tumor cells are occasionally arranged in clusters, while monocyte-lineage immune cells and spindle-shaped fibroblastic elements are visible in the background. C, Immunohistochemical staining for synaptophysin reveals strong positivity in the majority of cells, confirming the neuroendocrine neoplastic nature of most elements in the sample. D, Immunohistochemistry for CD45 highlights a subset of background cells as positive, consistent with their immune cell origin. Notably, neoplastic cells are negative for this marker.

To ascertain GEP-NET tumor cell content, we performed IHC analysis of 3 GEP-NET primary cultures, which were established following our standardized protocol (patients 10, 20, and an additional pNET primary culture without cell viability data), and we confirmed high tumor cell content of 99%, 90%, and 83%, respectively, with the remaining cells consisting of immune cells or fibroblasts (Table 2, Fig. 3).

**Table 2.** Immunohistochemical cell quantification

Patient ID	Tumor cells	Immune cells	Stromal cells
**10**	99%	1%	
**20**	90%	8%	2%
**Additional pNET**	83%	2%	15%

Abbreviations: ID, identification; pNET, pancreatic neuroendocrine tumor.

### Personalized drug screening platform

#### Targeted therapies

##### Signaling inhibitors (mechanistic target of rapamycin inhibitor everolimus, tyrosine kinase inhibitors cabozantinib and sunitinib, AKT inhibitor capivasertib, PI3K inhibitor alpelisib, CDK4/6 inhibitor ribociclib, HIF-2α inhibitor belzutifan)

We evaluated targeted (combination) therapies at clinically relevant concentrations or at concentrations close to the clinically relevant concentrations, including both established therapeutics for GEP-NETs (mTORi everolimus, TKIs sunitinib and cabozantinib) and emerging therapeutics (AKT inhibitor capivasertib, PI3K inhibitor alpelisib, CDK4/6 inhibitor ribociclib). The overall and individual primary culture therapy responses are listed in Table 3.

**Table 3.** Primary culture response to different substances and combination therapies, shown as the percentage of control DMSO (100%)

Drug		GEP-NET mean overall cell viability (%) (n) ±SD	GEP-NET mean cell viability by tumor type (%) (n) ±SD		GEP-NEC mean cell viability (%) (n) ±SD	Normal pancreatic tissue mean cell viability (%) (n) ±SD
			siNET	pNET		
Signaling inhibitors	Everolimus (10 nM)	76*^a^* (18) ± 0.16	*^b^* 72*^a^* (11) ± 0.15	82*^c^* (7) ± 0.16	69*^a^* (1) ± 0.18	**—**
	Capivasertib (5 µM)	67*^a^* (18) ± 0.2	73*^a^* (11) ± 0.13	*^b^* 60*^a^* (7) ± 0.25	72*^a^* (1) ± 0.17	**—**
	Capivasertib (10 µM)	63*^a^* (18) ± 0.2	64*^a^* (11) ± 0.12	60*^a^* (7) ± 0.29	51*^a^* (1) ± 0.1	—
	Everolimus (10 nM)/Capivasertib (5 µM)	55*^a^* (17) ± 0.18	57*^a^* (10) ± 0.12	53*^a^* (7) ± 0.24	48*^a^* (1) ± 0.11	—
	Alpelisib (5 µM)	68*^a^* (17) ± 0.15	66*^a^* (10) ± 0.09	70*^a^* (7) ± 0.2	66*^a^* (1) ± 0.15	—
	Alpelisib (5 µM)/Capivasertib (5 µM)	58*^a^* (17) ± 0.16	60*^a^* (10) ± 0.13	54*^a^* (7) ± 0.19	48*^a^* (1) ± 0.11	—
	Ribociclib (20 µM)	68*^a^* (9) ± 0.19	67*^a^* (6) ± 0.21	69*^a^* (3) ± 0.14	44*^a^* (1) ± 0.05	—
	Everolimus (10 nM)/ Ribociclib (20 µM)	58*^a^* (9) ± 0.13	61*^a^* (6) ± 0.1	52*^a^* (3) ± 0.17	43*^a^* (1) ± 0.07	—
	Alpelisib (5 µM)/ Ribociclib (20 µM)	50*^a^* (9) ± 0.1	54*^a^* (6) ± 0.08	43*^a^* (3) ± 0.11	32*^a^* (1) ± 0.06	—
	Sunitinib (0.5 µM)	82*^a^* (16) ± 0.17	80*^a^* (9) ± 0.15	85*^a^* (7) ± 0.19	47*^a^* (1) ± 0.11	—
	Cabozantinib (1 µM)	87*^a^* (3) ± 0.08	87 (2) ± 0.1	87 (1) ± 0.04	114 (1) ± 0.09	—
	Cabozantinib (2.5 µM)	87*^a^* (3) ± 0.1	89 (2) ± 0.13	84 (1) ± 0.03	86 (1) ± 0.1	—
	Cabozantinib (5 µM)	66*^a^* (19) ± 0.22	65*^a^* (11) ± 0.2	66*^a^* (8) ± 0.24	88 (1) ± 0.22	102 (1) ± 0.07
DDR inhibitors	Niraparib (5 µM)	78*^a^* (4) ± 0.06	76*^a^* (2) ± 0.07	78*^a^* (2) ± 0.06	73 (1) ± 0.16	—
	Adavosertib (10 µM)	72*^a^* (14) ± 0.15	75*^a^* (7) ± 0.12	70*^a^* (7) ± 0.18	76*^a^* (1) ± 0.14	—
	Berzosertib (1 µM)	91 (6) ± 0.35	*^b^* 79*^a^* (3) ± 0.43	102 (3) ± 0.22	19*^a^* (1) ± 0.15	—
	Berzosertib (10 µM)	35*^a^* (6) ± 0.32	*^b^* 20*^a^* (3) ± 0.28	47*^a^* (3) ± 0.30	7*^a^* (1) ± 0.01	—
Other targeted therapies, bisphosphonates	Belzutifan (4 µM)	99 (3) ± 0.1	103 (2) ± 0.1	91 (1) ± 0.2	—	—
	Belzutifan (5 µM)	96 (3) ± 0.08	101 (2) ± 0.07	89 (1) ± 0.01	57*^a^* (1) ± 0.11	—
	Belzutifan (20 µM)	89*^a^* (16) ± 0.19	97 (9) ± 0.15	*^b^* 81*^a^* (7) ± 0.19	—	—
	Entinostat (1 µM)	68*^a^* (17) ± 0.18	72*^a^* (10) ± 0.16	63*^a^* (7) ± 0.18	87 (1) ± 0.22	—
	Onalespib (1 µM)	53*^a^* (7) ± 0.23	64*^a^* (4) ± 0.13	*^b^* 36*^a^* (3) ± 0.25	66*^a^* (1) ± 0.21	—
	Onalespib (10 µM)	53*^a^* (7) ± 0.22	64*^a^* (4) ± 0.15	*^b^* 38*^a^* (3) ± 0.23	53*^a^* (1) ± 0.08	—
	PRI-724 (5 µM)	65*^a^* (14) ± 0.21	65*^a^* (8) ± 0.22	64*^a^* (6) ± 0.19	110 (1) ± 0.31	—
	PRI-724 (10 µM)	54*^a^* (13) ± 0.22	50*^a^* (7) ± 0.24	59*^a^* (6) ± 0.19	—	—
	G-1 (1 µM)	94 (11) ± 0.14	91 (6) ± 0.122	97 (5) ± 0.16	56*^a^* (1) ± 0.14	—
	G-1 (10 µM)	93 (11) ± 0.33	92 (6) ± 0.28	95 (5) ± 0.38	44*^a^* (1) ± 0.05	—
	Zoledronic acid (5 µM)	84*^a^* (16) ± 0.17	85*^a^* (9) ± 0.17	84*^a^* (7) ± 0.18	42*^a^* (1) ± 0.17	—
	Zoledronic acid (10 µM)	78*^a^* (16) ± 0.27	74*^a^* (9) ± 0.3	83*a* (7) ± 0.22	34*^a^* (1) ± 0.04	—
Chemotherapies	Temozolomide (100 µM)	89*^a^* (19) ± 0.2	*^b^* 85*^a^* (11) ± 0.22	95 (8) ± 0.15	88 (1) ± 0.25	—
	5-Fluorouracil (20 µM)	78*^a^* (15) ± 0.15	82*^a^* (8) ± 0.15	74*^a^* (7) ± 0.14	77 (1) ± 0.11	—
	Lurbinectedin (750 pM)	—	—	—	35*^a^* (1) ± 0.1	—
	Lurbinectedin (130 nM)	—	—	—	21*^a^* (1) ± 0.03	—
GLP-1/2 analogues	Semaglutide (100 nM)	94*^a^* (15) ± 0.16	91*^a^* (8) ± 0.13	98 (7) ± 0.18	93 (1) ± 0.25	—
	Semaglutide (1 µM)	99 (15) ± 0.21	103 (8) ± 0.25	*^b^* 93 (7) ± 0.15	89 (1) ± 0.26	—
	Teduglutide (10 nM)	95 (4) ± 0.2	96 (2) ± 0.25	93 (2) ± 0.14	—	—
	Teduglutide (50 nM)	105 (4) ± 0.26	120 (2) ± 0.27	91 (2) ± 0.12	—	—
Hormones	Estradiol (300 nM)	91*^a^* (4) ± 0.1	89*^a^* (3) ± 0.11	96 (1) ± 0.05	82 (1) ± 0.09	—
	Estradiol (1 µM)	95 (16) ± 0.17	96 (9) ± 0.15	95 (7) ± 0.2	72 (1) ± 0.12	—
	Progesterone (1 µM)	97 (14) ± 0.2	96 (7) ± 0.16	97 (7) ± 0.23	73 (1) ± 0.14	—
	Progesterone (10 µM)	96 (15) ± 0.26	105 (8) ± 0.14	*^b^* 87*^a^* (7) ± 0.33	78 (1) ± 0.3	—
	Estradiol (300 nM)/Progesterone (1 µM)	—	—	—	77 (1) ± 0.19	—
	Testosterone (30 nM)	88*^a^* (3) ± 0.06	87*^a^* (2) ± 0.07	89 (1) ± 0.03	76 (1) ± 0.31	—
	Testosterone (1 µM)	100 (14) ± 0.17	*^b^* 95 (7) ± 0.12	105 (7) ± 0.19	64*^a^* (1) ± 0.21	—
	DHEAS (1 µM)	93*^a^* (13) ± 0.18	95 (7) ± 0.15	91*^a^* (6) ± 0.21	—	—
	DHEAS (10 µM)	100 (14) ± 0.14	98 (7) ± 0.16	104 (7) ± 0.18	—	—
	Prolactin (20 nM)	100 (5) ± 0.11	99 (3) ± 0.11	102 (2) ± 0.11	92 (1) ± 0.03	—
	Oxytocin (1 nM)	94 (4) ± 0.11	93 (2) ± 0.1	96 (2) ± 0.12	90 (1) ± 0.1	—
	Oxytocin (10 nM)	103 (4) ± 0.21	115*^c^* (2) ± 0.23	*^b^* 92 (2) ± 0.07	81 (1) ± 0.19	—
	L-thyroxine (1 µM)	91*^a^* (10) ± 0.16	87*^a^* (6) ± 0.12	96 (4) ± 0.2	80 (1) ± 0.28	—
	L-thyroxine (10 µM)	93*^a^* (10) ± 0.16	95 (6) ± 0.11	91 (4) ± 0.2	47*^a^* (1) ± 0.06	—

Strong efficacy: cell viability decrease less than 50%, moderate efficacy: cell viability decrease 20% to 50%.

Abbreviations: DDR, DNA damage response; DHEAS, dehydroepiandrosterone sulfate; DMSO, dimethyl sulfoxide; GLP, glucagon-like peptide; NEC, neuroendocrine carcinoma; NET, neuroendocrine tumor; pNET, pancreatic NET; siNET, small intestinal NET.

^*a*^Statistically significant decrease in cell viability compared to control DMSO *P* less than .05.

^*b*^Statistically significantly higher efficacy of the listed therapy in the marked tumor type compared to the other tumor type.

^*c*^Statistically significant increase in cell viability compared to control DMSO *P* less than .05.

**Figure 4.**
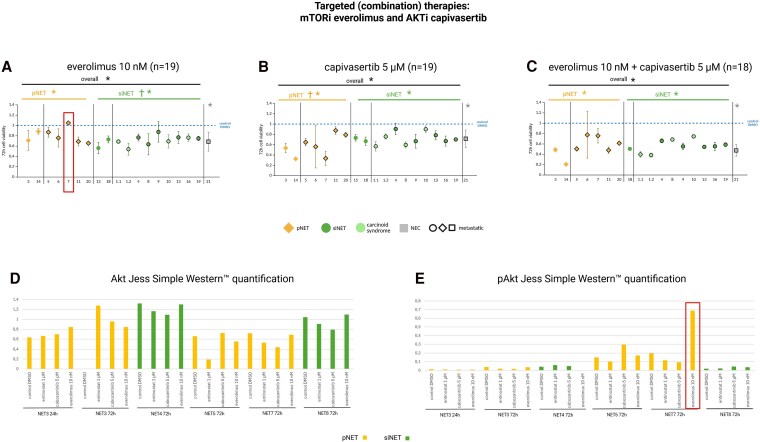
Cell viability of patient-derived gastroenteropancreatic neuroendocrine neoplasm (GEP-NEN) primary cultures after 72 hours’ incubation with clinically relevant concentrations of the targeted (combination) therapies A, everolimus 10 nM; B, capivasertib 5 µm; and C, everolimus 10 nM + capivasertib 5 µm. Primary cultures 1.1 and 1.2 are from the same patient. Pancreatic neuroendocrine tumors (pNETs) are shown in yellow, small intestinal NETs (siNETs) in green, siNETs with carcinoid syndrome in light green, and the GEP–neuroendocrine carcinoma (GEP-NEC) primary culture in gray. Metastatic tumors are outlined in black. Each cell viability experiment comprised 3 or 4 samples per drug concentration and patient. Mean values ± SD are shown. *Statistically significant decrease in cell viability compared to the control *P* less than .05. ns, not significant. †Statistically significantly higher efficacy of the listed therapy in the marked tumor type compared to the other tumor type. Statistical analysis was performed separately on the GEP-NET and GEP-NEC primary cultures. JESS Simple Western quantification of D, protein kinase B (Akt) and E, phospho (p)Akt, normalized to the respective total protein levels. pNETs in yellow, siNETs in green. Red box: high Akt activation (pAkt) in NET 7 after treatment with 10-nM everolimus, corresponding to the lacking everolimus-efficacy in the NET primary culture 7, as a potential mechanism of resistance.

**Figure 5.**
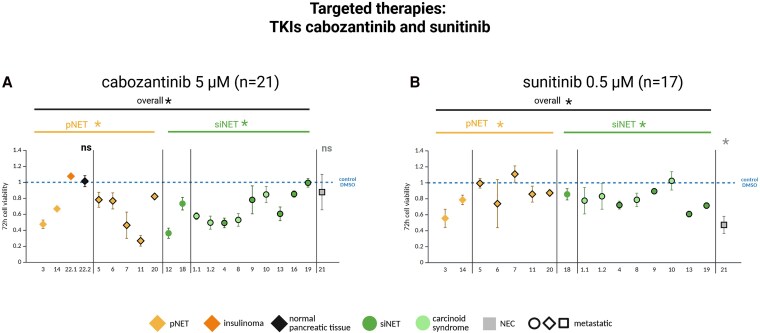
Cell viability of patient-derived gastroenteropancreatic neuroendocrine neoplasm (GEP-NEN) primary cultures, including one normal pancreatic tissue primary culture (corresponding tissue to the insulinoma primary culture), after 72 hours’ incubation with clinically relevant concentrations of the tyrosine kinase inhibitors A, cabozantinib 5 µm and B, sunitinib 0.5 µM. Primary cultures 1.1 and 1.2 as well as 22.1 and 22.2 are from the same patients, respectively. Pancreatic neuroendocrine tumors (pNETs) are shown in yellow, small intestinal NETs (siNETs) in green, siNETs with carcinoid syndrome in light green, the insulinoma in orange, normal pancreatic tissue in black, and the GEP–neuroendocrine carcinoma (GEP-NEC) in gray. Metastatic tumors are outlined in black. Each cell viability experiment comprised 3 or 4 samples per drug concentration and patient. Mean values ± SD are shown. *Statistically significant decrease in cell viability compared to the control *P* less than .05. ns, not significant. †Statistically significantly higher efficacy of the listed therapy in the marked tumor type compared to the other tumor type. Statistical analysis was performed separately on the GEP-NET, GEP-NEC, and normal pancreatic tissue primary cultures.

Overall, 10 nM everolimus (n = 18), 5 µM cabozantinib (n = 19), 5 µM capivasertib (n = 18), 5 µM alpelisib (n = 17), and 20 µM ribociclib (n = 9) demonstrated moderate, significant antitumor effects (mean cell viability 50%-76%) in GEP-NET primary cultures (Figs 4 and 5). Moderate antitumor efficacy was similarly found using the combination therapies everolimus/capivasertib (n = 17), alpelisib/capivasertib (n = 17), everolimus/ribociclib (n = 9), and alpelisib/ribociclib (n = 9). The combination therapies showed stronger efficacy than the single drugs, but no clear additive effects were found. In contrast, strong, significant efficacy was evident in the metastatic GEP-NEC primary culture using all the aforementioned combination therapies (mean cell viability 32%-48%).

Everolimus (10 nM) demonstrated a significantly higher efficacy in siNET (n = 11) compared to pNET (n = 7) primary cultures (mean cell viability siNET 72% vs pNET 82%), while the opposite was found using 5 µM capivasertib (mean cell viability pNET 60% vs siNET 73%; Fig. 4A and 4B).

Automated Western blot data showed relatively low levels of activated pAkt in representative GEP-NET primary cultures after incubation with the control (DMSO vehicle) or different targeted therapies (everolimus, cabozantinib, entinostat). Exceptionally, in NET number 7, everolimus (10 nM) led to strong Akt activation, compared to the control (Fig. 4C) and, interestingly, cell viability data showed no efficacy of everolimus in the corresponding primary culture (see Fig. 4A), indicating everolimus-induced Akt upregulation as a potential resistance mechanism.

The multi-TKI sunitinib (0.5 µM: n = 16) demonstrated overall low antitumor efficacy in our in vitro model (mean cell viability 82%), but strong efficacy in the metastatic GEP-NEC primary culture (mean cell viability 47%; Fig. 5B). Also, the HIF-2α inhibitor belzutifan showed overall no effects at low, clinically relevant concentrations (4-5 µM; n = 3) and low antitumor effects using higher concentrations (20 µM; n = 16), with significantly higher efficacy in pNETs compared to siNETs (mean cell viability 81% vs 97%).

##### DNA damage response inhibitors (PARP inhibitor niraparib, WEE1 inhibitor adavosertib, ataxia telangiectasia and Rad3-related inhibitor berzosertib)

**Figure 6.**
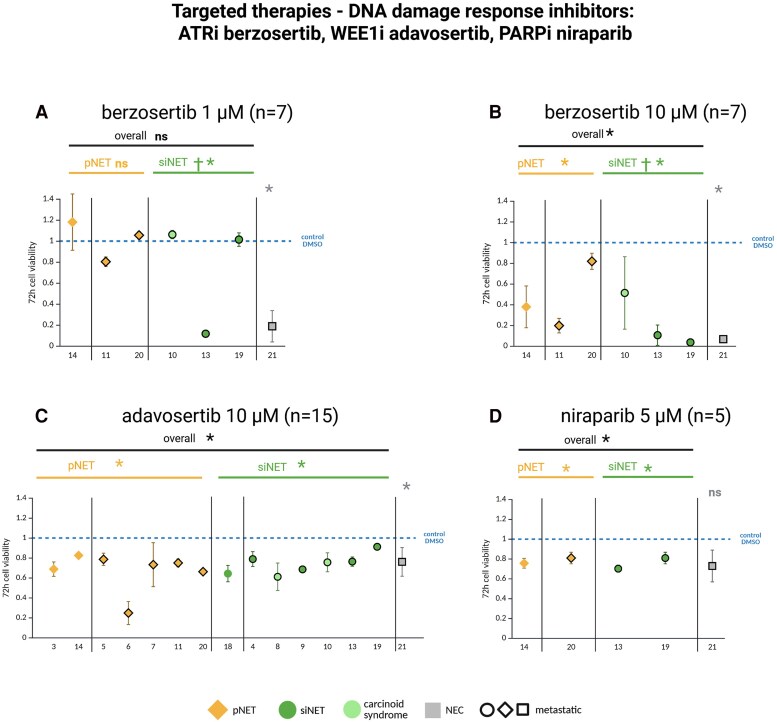
Cell viability of patient-derived gastroenteropancreatic neuroendocrine neoplasm (GEP-NEN) primary cultures after 72 hours’ incubation with DNA damage response inhibitors A, berzosertib 1 µm (clinically relevant concentration) and B, 10 µm; C, adavosertib 10 µm; and D, niraparib 5 µm (clinically relevant concentration). Primary cultures 1.1 and 1.2 are from the same patient. Pancreatic neuroendocrine tumors (pNETs) are shown in yellow, small intestinal NETs (siNETs) in green, siNETs with carcinoid syndrome in light green, and the GEP–neuroendocrine carcinoma (GEP-NEC) primary culture in gray. Metastatic tumors are outlined in black. Each cell viability experiment comprised 3 or 4 samples per drug concentration and patient. Mean values ± SD are shown. *Statistically significant decrease in cell viability compared to the control *P* less than .05. ns, not significant. †Statistically significantly higher efficacy of the listed therapy in the marked tumor type compared to the other tumor type. Statistical analysis was performed separately on the GEP-NET and GEP-NEC primary cultures.

DNA damage response (DDR) inhibitors (PARP inhibitor niraparib, WEE1 inhibitor adavosertib, ataxia telangiectasia and Rad3-related [ATR] inhibitor berzosertib) were also evaluated, and showed overall moderate (5 µM niraparib: n = 4, 10 µM adavosertib: n = 14; mean cell viability 72%-78%) to strong (10 µM berzosertib: n = 6, mean cell viability 35%) antitumor effects in GEP-NET primary cultures (Fig. 6). Particularly strong efficacy of berzosertib was found in the GEP-NEC primary culture at clinically relevant (1 µM; mean cell viability 19%, Fig. 6A) or higher concentrations (10 µM; mean cell viability 7%; Fig. 6B). Additionally, berzosertib demonstrated significantly higher efficacy in siNETs compared to pNETs (mean cell viability 1 µM berzosertib 79% vs 102%, 10 µM berzosertib 20% vs 47%; see Fig. 6A).

##### Other targeted therapies and epigenetic modifiers (Hsp90 inhibitor onalespib, HDAC inhibitor entinostat, Wnt/β-catenin/CBP inhibitor PRI-725)

Novel targeted therapies currently investigated in clinical trials were additionally tested (Hsp90 inhibitor onalespib 1-10 µM: n = 7, HDAC inhibitor entinostat 1 µM: n = 17, Wnt/β-catenin/CBP inhibitor PRI-724 5-10 µM: n = 14), all showing overall moderate antitumor effects (mean cell viability 53%-68%). Strikingly, onalespib at clinically relevant (1 µM) and higher concentrations (10 µM) showed strong efficacy in pNETs and significantly stronger efficacy in pNETs compared to siNETs (mean cell viability 36%-38% vs 64%) (see Table 3).

#### Chemotherapeutic agents

**Figure 7.**
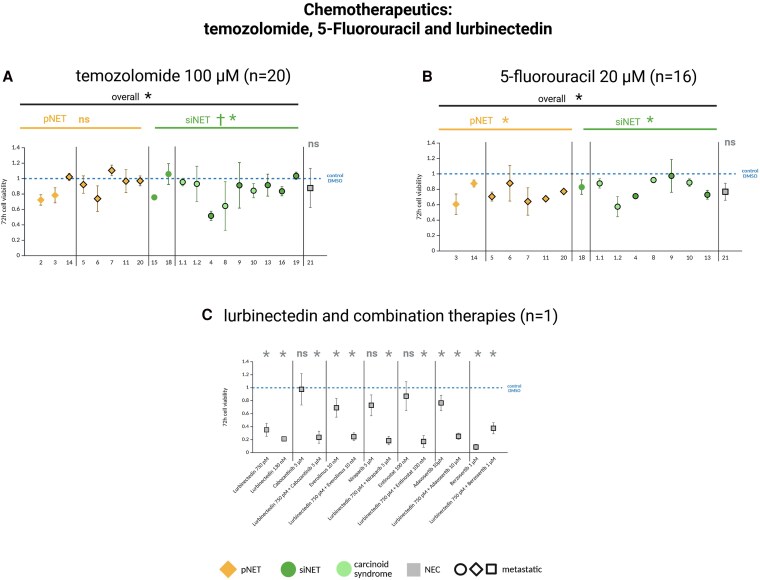
Cell viability of patient-derived gastroenteropancreatic neuroendocrine neoplasm (GEP-NEN) primary cultures after 72 hours’ incubation with clinically relevant concentrations of the chemotherapeutics A, temozolomide 100 µm; B, 5-fluorouracil 20 µm; and C, lurbinectedin alone and in combination with different targeted therapies (tested in the GEP–neuroendocrine carcinoma [GEP-NEC] primary culture 21). Primary cultures 1.1 and 1.2 are from the same patient. Pancreatic neuroendocrine tumors (pNETs) are shown in yellow, small intestinal NETs (siNETs) in green, siNETs with carcinoid syndrome in light green, and the GEP-NEC primary culture in gray. Metastatic tumors are outlined in black. Each cell viability experiment comprised 3 or 4 samples per drug concentration and patient. Mean values ± SD are shown. *Statistically significant decrease in cell viability compared to the control *P* less than .05. ns, not significant. †Statistically significantly higher efficacy of the listed therapy in the marked tumor type compared to the other tumor type. Statistical analysis was performed separately on the GEP-NET and GEP-NEC primary cultures.

Chemotherapeutics established in GEP-NETs (temozolomide, 5-fluorouracil) or in small cell lung cancer (lurbinectedin) were evaluated (Fig. 7). Temozolomide monotherapy displayed overall low efficacy (100 µM: n = 19, mean cell viability 89%), while 5-fluorouracil showed overall moderate efficacy (20 µM: n = 15, mean cell viability 78%).

Lurbinectedin, currently tested only in the GEP-NEC primary culture (n = 1), led to strong, significant cell viability decrease at clinically-relevant concentrations (130 nM: mean cell viability 21%; Fig. 7C). Lurbinectedin combined with different targeted therapies did not increase efficacy.

#### Repurposed drugs

The bone-targeted agent/bisphosphonate zoledronic acid (5 µM, 10 µM) showed low to moderate antitumor effects in GEP-NET primary cultures (n = 16, mean cell viability 78%-84%) but particularly strong significant efficacy in the GEP-NEC primary culture (mean cell viability 34%-42%).

### Personalized risk assessment platform

#### Glucagon-like peptide-1/Glucagon-like peptide-2 analogues

The GLP-2 analogue teduglutide, approved for therapy of short bowel syndrome with intestinal failure (19), was evaluated in GEP-NET primary cultures and showed overall no effects, particularly no tumor-promoting effects, after prolonged incubation times of 7 days (n = 4).

The GLP-1 analogue semaglutide, approved for therapy of type 2 diabetes mellitus and obesity, showed overall low to no effects (n = 15) in GEP-NETs, but higher efficacy in pNETs compared to siNETs (mean cell viability 93% vs 103%) at 1 µM. Importantly, no tumor-promoting effects of semaglutide were found.

#### Sex hormones

We also include our risk assessment data evaluating elevated sex hormone levels that was partially previously published (16). We now include a larger patient cohort (n = 16) and complemented the results with automated Western blot data.

**Figure 8.**
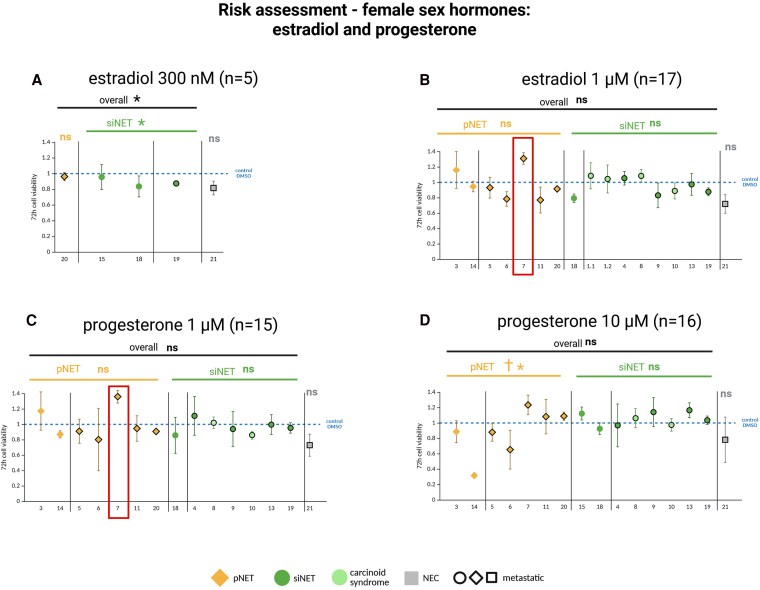
Cell viability of patient-derived gastroenteropancreatic neuroendocrine neoplasm (GEP-NEN) primary cultures after 72 hours’ incubation with female sex hormones A, estradiol 300 nM (clinically relevant concentration) and B, 1 µm; C, progesterone 1 µm (clinically relevant concentration); and D, 10 µm. Primary cultures 1.1 and 1.2 are from the same patient. Pancreatic neuroendocrine tumors (pNETs) are shown in yellow, small intestinal NETs (siNETs) in green, siNETs with carcinoid syndrome in light green, and the GEP–neuroendocrine carcinoma (GEP-NEC) primary culture in gray. Metastatic tumors are outlined in black. Red box: Tumor-promoting effects both of estradiol and progesterone in NET 7 (male patient). Each cell viability experiment comprised 3 or 4 samples per drug concentration and patient. Mean values ± SD are shown. *Statistically significant decrease in cell viability compared to the control *P* less than .05. ns, not statistically significant. †Statistically significantly higher efficacy of the listed therapy in the marked tumor type compared to the other tumor type. Statistical analysis was performed separately on the GEP-NET and GEP-NEC primary cultures.

Elevated female sex hormone levels may be found in GEP-NET patients during pregnancy or hormone replacement therapy. We found overall no tumor-promoting effects of estradiol (1 µM; n = 16), progesterone (1 µM, 10 µM; n = 15), or the combination of both (n = 1) in GEP-NETs or GEP-NEC primary cultures (Fig. 8). However, heterogeneous tumor responses were observed: For example, in one metastatic pNET (NET 7) both estradiol and progesterone induced tumor-promoting effects (see Fig. 8B and 8C).

Based on the estrogen data, we also evaluated the G protein–coupled estrogen receptor agonist G-1 and found overall no effects in GEP-NETs, but moderate to strong antitumor effects in the GEP-NEC primary culture (mean cell viability 44%-56%).

The androgens testosterone and dehydroepiandrosterone sulfate (DHEAS) similarly showed no tumor-promoting effects, but overall even low, significant antitumor efficacy at clinically relevant concentrations. Moreover, higher concentrations of testosterone (1 µM) demonstrated significantly higher efficacy in siNETs compared to pNETs (mean cell viability 95% vs 105%).

**Figure 9.**
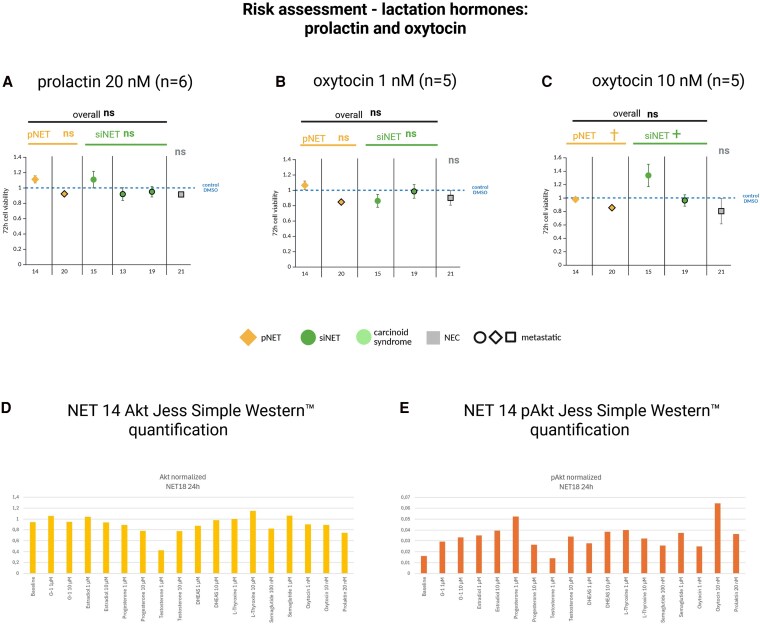
Cell viability of patient-derived gastroenteropancreatic neuroendocrine neoplasm (GEP-NEN) primary cultures after 72 hours’ incubation with clinically relevant concentrations of the lactation hormones A, prolactin 20 nM and B, oxytocin 1 nM; and C, 10 nM. Primary cultures 1.1 and 1.2 are from the same patient. Pancreatic neuroendocrine tumors (pNETs) are shown in yellow, small intestinal NETs (siNETs) in green, siNETs with carcinoid syndrome in light green, and the GEP–neuroendocrine carcinoma (GEP-NEC) primary culture in gray. Metastatic tumors are outlined in black. Each cell viability experiment comprised 3 or 4 samples per drug concentration and patient. Mean values ± SD are shown. *Statistically significant decrease in cell viability compared to the control *P* less than .05. ns, not statistically significant. †Statistically significantly higher efficacy of the listed therapy in the marked tumor type compared to the other tumor type. Statistical analysis was performed separately on the GEP-NET and GEP-NEC primary cultures. JESS Simple Western quantification of D, protein kinase B (Akt) and E, phospho (p)Akt in NET 14, normalized to the respective total protein levels, after treatment with different hormones and semaglutide.

No relevant Akt activation or inhibition was found after treatment using sex hormones, G-1, or semaglutide (Fig. 9D).

#### Lactation hormones

We assessed hormones involved in lactation (prolactin n = 5, oxytocin n = 4) to perform a risk assessment of breastfeeding in GEP-NET patients (see Fig. 9). Overall, both hormones showed no antitumor or tumor-promoting effects, but importantly, in siNETs (n = 2), oxytocin at high concentrations (10 nM) demonstrated significant tumor-promoting effects (Fig. 9C).

#### Thyroid hormones

L-thyroxine was evaluated as the synthetic form of the thyroid hormone thyroxine to assess the risk of elevated thyroid hormone levels on GEP-NETs (eg, occurring with manifest/subclinical hyperthyroidism). Interestingly, l-thyroxine (1 µM, 10 µM) led to overall low, but significant, cell viability decreases in GEP-NETs (n = 10) while the higher concentrations (10 µM) strongly decreased cell viability in the GEP-NEC (mean cell viability 47%).

## Discussion

Given the need for novel and effective therapies and the absence of precision therapies in GEP-NENs to date, we have established a standardized platform for personalized drug screening and risk assessment using patient-derived GEP-NEN primary cultures. We systematically evaluated 27 different agents, including established and putative novel targeted therapies such as kinase signaling inhibitors, DDR inhibitors, chemotherapeutic agents, and repurposed agents. Drug screening of many different drugs is ongoing. The patient-derived GEP-NEN platform can additionally be used for personalized risk assessment by studying, for example, the effects of different hormones on tumor growth (eg, GLP-1/GLP-2 analogues, sex hormones, or thyroid hormones). We observed both significant group effects and pronounced interindividualized heterogeneity including differential drug responses between pNETs and siNETs as well as between GEP-NETs and a single GEP-NEC, respectively. All primary culture findings were correlated with clinical characteristics and tumor biology of the individual patients, supporting the translational potential of this approach.

### Personalized drug screening platform

We found moderate to strong efficacy of most targeted therapies alone and in combination in GEP-NETs with significantly higher efficacy of some substances in pNETs (AKT inhibitor capivasertib, high-dose HIF-2α inhibitor belzutifan, Hsp90 inhibitor onalespib) and of other substances in siNETs (mTOR inhibitor everolimus, ATR inhibitor berzosertib).

Everolimus is US Food and Drug Administration (FDA) and European Medicines Agency (EMEA) approved for treatment both of pNETs and extrapancreatic NETs (siNETs and lung-NETs), based on results from the RADIANT-3 and RADIANT-4 trials (10, 11). Our automated Western blot analysis showed strong Akt activation in one pNET after treatment with everolimus and, correspondingly, no efficacy of everolimus in the same primary culture (NET 7). Other GEP-NET primary cultures, which responded well to everolimus, showed no relevant Akt activation. Interestingly, NET 7 was not previously treated with everolimus but received lanreotide and peptide receptor radionuclide therapy, potentially suggesting a preexisting everolimus resistance. Previous studies have shown that Akt feedback activation can attenuate everolimus response while combination therapies with PI3K/Akt-inhibiting drugs could in turn increase everolimus sensitivity (20-26). Therefore, we have now evaluated the combination therapy of everolimus with the Akt inhibitor capivasertib, an officially approved therapy for certain types of breast cancer, demonstrating increased efficacy compared to the single therapies in GEP-NET primary cultures.

The TKI sunitinib is FDA and EMEA approved for treatment of pNETs, based on results from a phase 3 trial (12). The TKI cabozantinib is FDA and EMEA approved for pancreatic and extrapancreatic NETs based on results from the CABINET trial (NCT03375320) (27). Both substances demonstrated low to moderate efficacy in the GEP-NET primary cultures without differences in efficacy between pNET and siNET. Similarly, in the randomized phase 3 trial, cabozantinib showed no survival difference between progressive pNET or extrapancreatic NET (27). In contrast to the low efficacy of sunitinib in GEP-NETs, strong efficacy was found in the GEP-NEC primary culture. However, a previous study found significantly better progression-free survival and overall survival of patients receiving sunitinib therapy with NET G1-3 than in patients with NEC G3 (28). This may be explained by the generally worse prognosis of GEP-NEC (4-6, 29) and, moreover, tumor response of GEP-NECs could differ individually. Other clinical studies have also identified both responders and nonresponders to sunitinib (30). Additionally, higher efficacy of targeted combination therapies (everolimus/capivasertib, alpelisib/capivasertib, everolimus/ribociclib, alpelisib/ribociclib) was found in the GEP-NEC primary culture (mean viability 32%-48%; strongest combination therapy: alpelisib/ribociclib). Nevertheless, more GEP-NEC primary culture data are needed for definitive conclusions. In GEP-NET primary cultures, these combination therapies showed stronger efficacy than the single drugs, but no clear additive effects.

Belzutifan has been approved by the FDA and EMEA in patients with von-Hippel Lindau syndrome and renal cell carcinoma, hemangioblastoma, or pNETs based on results from the LITESPARK-004 study (NCT03401788) (31, 32), as well as in patients with metastatic renal cell carcinoma based on results from the LITESPARK-005 study (NCT04195750) (33). Recently, belzutifan also received FDA approval as the first systemic oral therapy for advanced pheochromocytoma and paraganglioma based on results from the LITESPARK-015 study (NCT04924075). We found low antitumor effects of high-dose belzutifan with stronger efficacy in pNETs compared to siNETs.

High concentrations of the ATR inhibitor berzosertib showed the strongest overall antitumor efficacy in GEP-NET primary cultures (mean cell viability 35%) and the GEP-NEC primary culture (mean cell viability 7%). Berzosertib is currently being investigated in combination with lurbinectedin in NEC patients (NCT04802174). Strikingly, the chemotherapeutic agent lurbinectedin, FDA approved for small cell lung cancer, demonstrated the second strongest efficacy at clinically relevant concentrations (130 nM) in the GEP-NEC primary culture (mean cell viability 21%).

The radiosensitizer onalespib is being investigated in phase 1/2 clinical trials for breast and lung cancer in combination with other therapies, but concerns regarding tolerability and limited efficacy have partly been found (34, 35). In contrast, in NETs, onalespib potentiated the effects of ^177^Lu-DOTATATE therapy in mouse NET xenografts and showed a favorable toxicity profile (36). Particularly in our pNET primary cultures, onalespib showed strong, significant efficacy at clinically relevant concentrations (mean cell viability 36%).

Chemotherapeutics temozolomide and 5-fluorouracil showed only low to moderate efficacy in GEP-NET primary cultures, with increased efficacy of temozolomide in siNETs compared to pNETs.

Additionally, we explored repurposed therapeutics for other indications and found low to moderate antitumor effects of the bone-targeted agent zoledronic acid, with particularly strong efficacy in the GEP-NEC primary culture. Zoledronic acid has previously demonstrated in vitro and in vivo antitumor effects in multiple cancer types (23, 37-40) including NECs (41), but to our knowledge no data are as yet available on GEP-NETs.

### Personalized risk assessment platform

Another important area of application of our patient-derived GEP-NEN platform lies in the possibility to perform individual risk assessment, for example, regarding the effects of different hormones on tumor growth. GLP1/2 analogues semaglutide and teduglutide showed overall no tumor-promoting effects. Low antitumor efficacy of semaglutide was found, with slightly stronger efficacy in pNETs compared to siNETs.

We further show that elevated sex hormone levels (eg, found in patients with GEP-NETs during pregnancy or hormone replacement therapy), induced no tumor-promoting effects. These data were partly previously published (16), but now confirmed in a larger cohort of GEP-NETs (n = 16) and a GEP-NEC (n = 1). Importantly, heterogeneous individual tumor response can be observed, as demonstrated in a metastatic pNET primary culture from a male patient reacting with tumor-promoting effects following incubation with female sex hormones.

Androgens (testosterone and DHEAS; n = 14) or the synthetic thyroid hormone l-thyroxine (n = 10) showed no tumor-promoting effects. Lactation hormones prolactin (n = 5) and oxytocin (n = 4) showed no tumor-promoting effects at doses close to the clinically-relevant concentrations. However, oxytocin at supraphysiological concentrations demonstrated significant tumor-promoting effects in 2 siNETs. To our knowledge, there are no studies evaluating the effects of lactation on NET tumor growth to date. However, a previous study has shown that diagnosis of other cancer types, excluding breast or ovarian cancer, during pregnancy or lactation did not increase the risk of cause-specific death (42).

### Limitations

A limitation of the patient-derived primary culture platform is that it allows assessment of tumor cell death but does not capture disease stabilization, which represents a clinically relevant component of therapeutic response (disease control rate) in vivo. Therefore, these in vitro data can be interpreted and used fully only once the in vivo effects of the drugs in patients can be directly correlated with the primary culture results. This will be subject of future studies. Another limitation of our study is that a 2-dimensional primary culture model cannot fully represent the in vivo situation, in which tumor cells may be less susceptible to the treatment effects due to the 3-dimensional structure of the tumor. This may be overcome by 3-dimensional organoid models, but only in vivo models will also allow for the evaluation of drug toxicity.

Additionally, tumor composition of different primary cultures varies. We confirmed high tumor cell content of more than 80% in 3 representative GEP-NET primary cultures. However, the fact that nonneoplastic cells (eg, immune cells, fibroblasts) are present in our primary cultures may be an advantage since patient tumors are also heterogeneous and primary cultures can therefore reflect tumor cell interactions, in contrast to cell line models, as previously reviewed (43).

### Conclusions

Overall, precision medicine is not yet established in GEP-NENs and is particularly difficult in these tumors, which do not show high rates of molecular alterations (44). The strength of our platform lies in the possibility of personalized drug screening, allowing for a correlation of the individual tumor primary culture response with clinical patient characteristics, tumor biology, and kinase signaling data. Results from freshly surgery-derived patient GEP-NENs are generated using a standardized protocol and are quickly available after resection of the primary tumor or metastasis within 2 weeks. This potentially enables a prediction of effective/less effective therapies as well as therapy recommendations on a case-by-case basis. Additionally, the personalized risk assessment platform allows for individual recommendations with regard to conditions such as obesity treatment, hyperthyroidism, pregnancy, or breastfeeding in patients with GEP-NENs, history of GEP-NENs, or genetic predispositions. Furthermore, GEP-NEN sequencing is planned for future studies that will enable the correlation of genetic status with individual drug response in terms of true precision medicine, as already established and standardized in our pheochromocytoma/paraganglioma patient-derived tumor platform (15, 16, 23, 43).

## Data Availability

The data supporting the findings of this study are available within the paper and its supplemental information (18).

## Abbreviations

Abs: antibodies
Akt: protein kinase B
DDR: DNA damage response
DHEAS: dehydroepiandrosterone sulfate
DMSO: dimethyl sulfoxide
EMEA: European Medicines Agency
FDA: US Food and Drug Administration
GEP-NENs: gastroenteropancreatic neuroendocrine neoplasms
GLP: glucagon-like peptide
IHC: immunohistochemical
mTORi: mechanistic target of rapamycin inhibitor
NEC: neuroendocrine carcinoma
NEN: neuroendocrine neoplasm
NET: neuroendocrine tumor
pNET: pancreatic neuroendocrine tumor
siNET: small intestinal neuroendocrine tumor
SSTR2: somatostatin receptor 2
TKIs: tyrosine kinase inhibitors
